# Beyond the right ventricle: assessment of the left ventricular function in pulmonary hypertension

**DOI:** 10.3389/fmed.2026.1832363

**Published:** 2026-04-30

**Authors:** Yilin Xie, Tianyu Wang, Yingjie Tan, Jiang Li

**Affiliations:** Department of Cardiovascular Medicine, The Second Xiangya Hospital of Central South University, Changsha City, China

**Keywords:** cardiac magnetic resonance, echocardiography, interventricular septum, left ventricle, multimodality imaging, pulmonary hypertension, ventricular interdependence

## Abstract

Pulmonary hypertension (PH) is a pathological condition characterized by elevated pressure in the pulmonary artery, often accompanied by right ventricular (RV) dysfunction. Nevertheless, the significance of left ventricular (LV) function in PH is being increasingly acknowledged, especially in the context of ventricular interdependence and the interactions between the ventricles. This review comprehensively examines the anatomical, physiological, and pathophysiological foundations of LV function assessment in patients with PH while summarizing advancements in evaluating LV function within this context. Specifically, the evaluation of LV diastolic function, systolic function, alterations in interventricular septal morphology, and the presence of ventricular dyssynchrony play crucial roles in the comprehensive understanding of left ventricular dynamics and differentiating pre-capillary PH from post-capillary PH. Despite the availability of various assessment parameters, limitations, including the lack of standardized criteria and insufficient specificity, continue to prevail. Future research should concentrate on developing specific indicators, establishing unified assessment standards, and integrating multimodal imaging techniques to evaluate LV function comprehensively, aiming to enhance prognosis and optimize treatment strategies for PH patients.

## 1 Introduction

Pulmonary hypertension (PH) constitutes a complex pathological state primarily characterized by elevated pulmonary artery pressure and right ventricular dysfunction (1). According to the latest 2022 ESC/ERS guidelines, PH is hemodynamically defined as a mean pulmonary arterial pressure (mPAP) > 20 mmHg at rest (2). Current estimates indicate that PH affects approximately 1% of the global population, with prevalence increasing to 10% among individuals aged 65 and older (3). Although right ventricular (RV) dysfunction is a prominent feature of PH, the significance of left ventricular (LV) function is recognized increasingly in this condition. The close anatomical and physiological relationship between the left and right ventricles indicates that abnormalities in the function of one ventricle can directly influence the performance of the other through the interventricular septum. In the context of PH, persistent pressure overload on the RV adversely impacts LV filling and contraction capabilities due to septal displacement and pericardial constraints. Consequently, an accurate assessment of LV function is essential for comprehending the pathophysiological mechanisms underlying PH, optimizing therapeutic strategies, and enhancing patient outcomes. Crucially, the clinical interpretation of LV parameters must be phenotype-driven to ensure diagnostic accuracy. In pre-capillary PH, LV abnormalities primarily manifest as mechanical underfilling and compression secondary to RV overload. Conversely, in post-capillary PH, intrinsic LV dysfunction or remodeling serves as the primary driver of elevated pulmonary pressures. This article critically evaluates the advancements in the assessment of LV function in PH, emphasizing a phenotype-specific framework to identify which parameters—such as LVEDP, LVFP, and the eccentricity index—are most effective for differentiating between pre-capillary and post-capillary states. By identifying existing limitations, we propose future research directions to enhance clinical decision-making across the diverse spectrum of PH.

## 2 Anatomy and physiology

**Figure 1:**
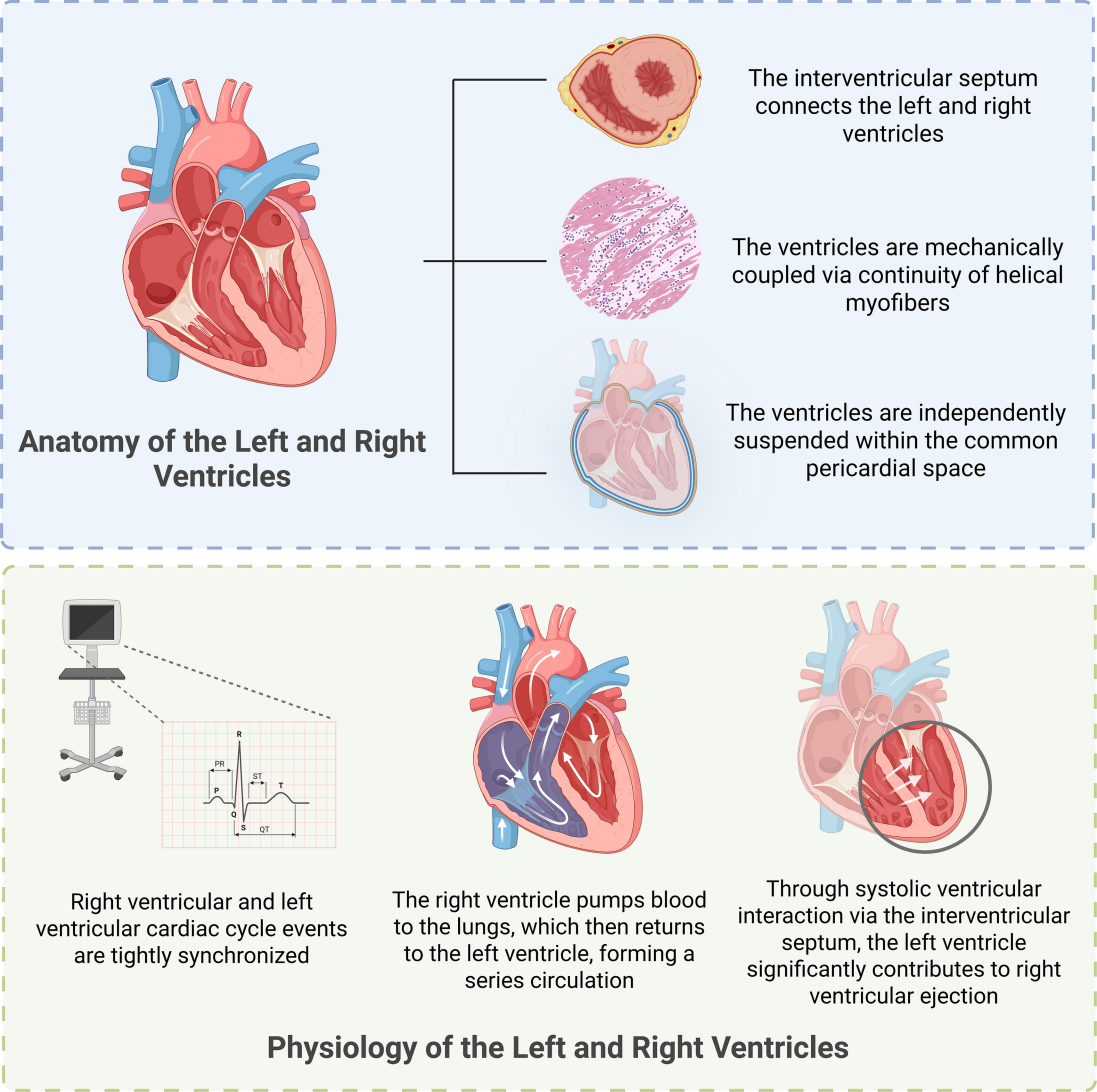
The anatomy and physiology of the left and right ventricles. Created using BioRender.com.

The left ventricle and right ventricle exhibit a close relationship regarding anatomical structure and physiological function. Anatomically, The LV is connected to the RV by the interventricular septum, has epicardial fibers that surround each other, and shares a (non-acute) expandable pericardium (4–6) (Figure 1). In the physiological context, the events of the cardiac cycle for both the RV and LV, such as the onset of contraction and the filling of the aorta and pulmonary artery, are closely synchronized (7). The RV functions as a volume-loaded pump, ejecting systemic venous return into the pulmonary circulation for gas exchange, which subsequently travels to the LV via the pulmonary veins and left atrium (LA), ultimately facilitating a continuous circulation. This phenomenon results in equivalent stroke volumes between the two ventricles (8–10). Moreover, due to the interventricular contraction interaction, the LV considerably contributes to RV ejection: it is estimated that 20–40% of the RV’s energy supply (systolic pressure and volume outflow) is derived from LV ejection in healthy individuals (8, 11) (Figure 1). These characteristics highlight a crucial aspect of LV-RV physiology—ventricular interdependence. Under adverse loading conditions, asynchronous cardiac cycles, and ventricular failure, both ventricles may mutually influence each other’s filling and contraction capabilities, thereby leading to ventricular dysfunction and unfavorable ventricular-ventricular interactions.

## 3 Pathophysiology

PH results in left ventricular dysfunction owing to the interdependence between the ventricles (12). The term “ventricular-ventricular interaction” delineates how the function, geometry, and events of one ventricle influence the other (7). Within the pathological context of PH, the right ventricle undergoes chronic pressure overload, whereby severely elevated pressures can adversely affect left ventricular function through ventricular interdependence, exacerbating symptoms (13, 14). Indeed, patients with PH exhibit diminished systolic and diastolic functions of the left ventricle, which can be elucidated as follows (15).

First, the relative inextensibility of the pericardium leads to dilation of the right ventricle under pressure overload, thereby exerting pressure on the left ventricle through the interventricular septum. This impairs left ventricular filling and exacerbates the ventricular-ventricular interaction (7). This phenomenon is characterized by the paradoxical movement of the interventricular septum, a “D” shape of the left ventricle, and an increased left ventricular eccentricity index (16). Furthermore, these secondary geometric alterations in the left ventricle demonstrate a linear correlation with cardiac output, resulting in reductions in stroke volume and coronary perfusion (4, 17–19). Moreover, even in the absence of intrinsic left ventricular disease, mild idiopathic pulmonary arterial hypertension (IPAH) can impair left ventricular diastolic compliance (13). Echocardiographic assessment elucidates this measurement, revealing impaired left ventricular relaxation as a common abnormality among patients with PH (20). Furthermore, the reduction in cardiac output resulting from right ventricular failure contributes to inadequate filling of the left atrium and left ventricle due to indirect circulatory interactions (16). The asynchrony between the ventricles, accompanied by post-systolic shortening is another contributing factor to adverse ventricular interaction in pulmonary arterial hypertension (21). This phenomenon correlates with escalating pulmonary arterial pressure (5, 22). PH also results in right ventricular dysfunction, characterized by prolonged right ventricular contraction (potentially extending beyond the pulmonary valve closure time) and a significant leftward shift of the interventricular septum, leading to shorter ejection time and reduced stroke volume. This contraction persists into the early diastole phase of the left ventricle, contributing to inadequate left ventricular filling (7, 10, 17, 23, 24).

**Figure 2:**
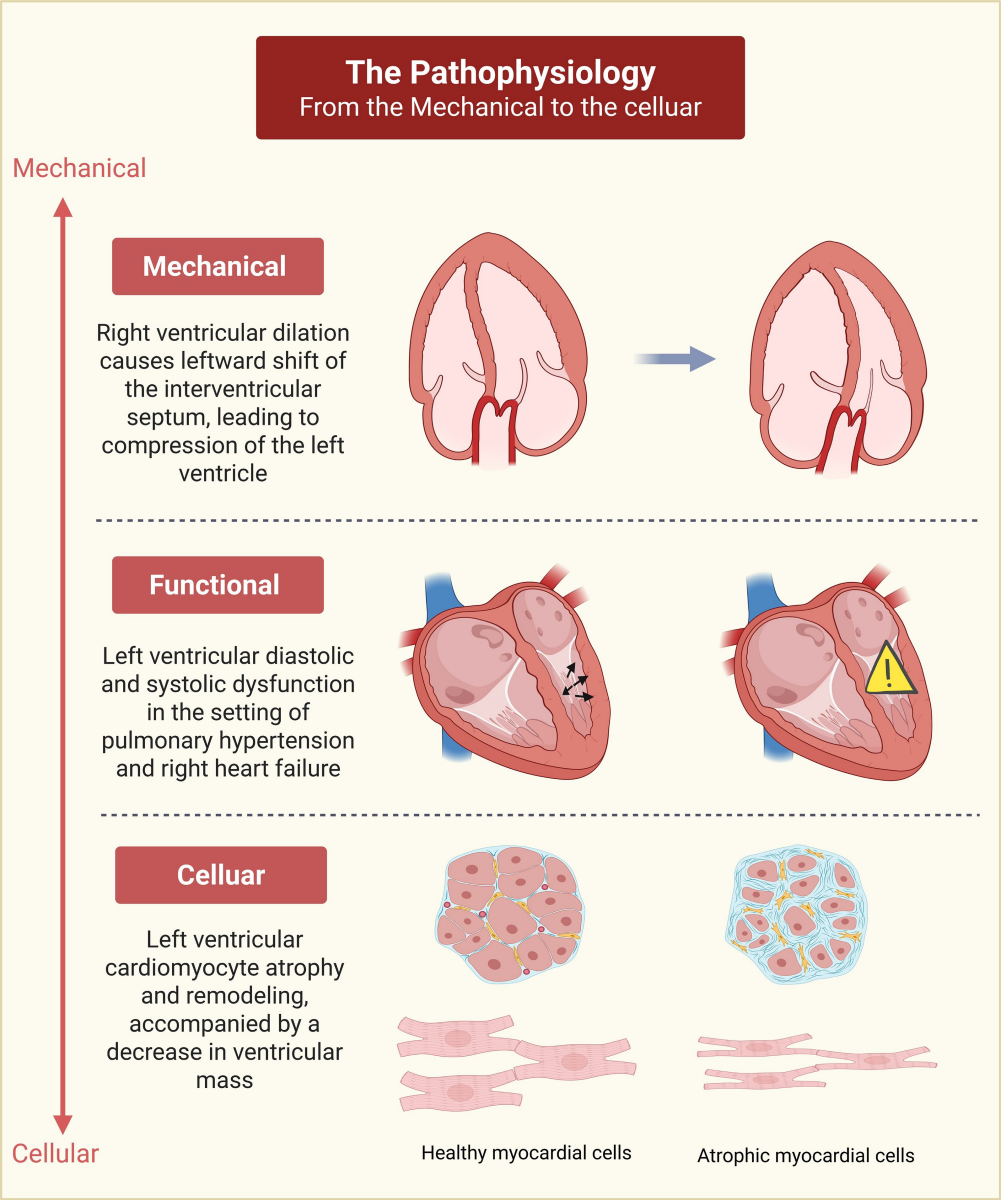
Hierarchical pathophysiology of LV dysfunction in PH: From mechanical to cellular levels. Created using BioRender.com.

As the disease advances toward end-stage PH, these responses may induce atrophic remodeling of the left ventricle (25, 26). At the molecular level, this adaptation involves a shift toward a fetal gene expression profile and the downregulation of major contractile and regulatory proteins (27). Specifically, the significant reduction in contractile proteins, such as myosin, alongside decreased sarcomeric protein phosphorylation, severely impairs intrinsic contractile function (28). Concurrently, enhanced autophagic activity acts as a primary driver of cardiomyocyte degradation, which is further exacerbated by myocardial hypoxia and ischemia secondary to right ventricular failure (29, 30). These intrinsic changes ultimately culminate in marked decreases in cardiomyocyte cross-sectional area (CSA) (28) alongside reductions in myocardial mass (27, 29). Consequently, it is essential to accurately assess left ventricular function, providing insights into the ventricular-ventricular interactions and the overall functioning of both the left and right hearts in the context of PH and right ventricular failure, thereby enhancing outcomes for patients and effectively guiding treatment strategies (Figure 2).

## 4 Assessment of left ventricular diastolic function

**Figure 3:**
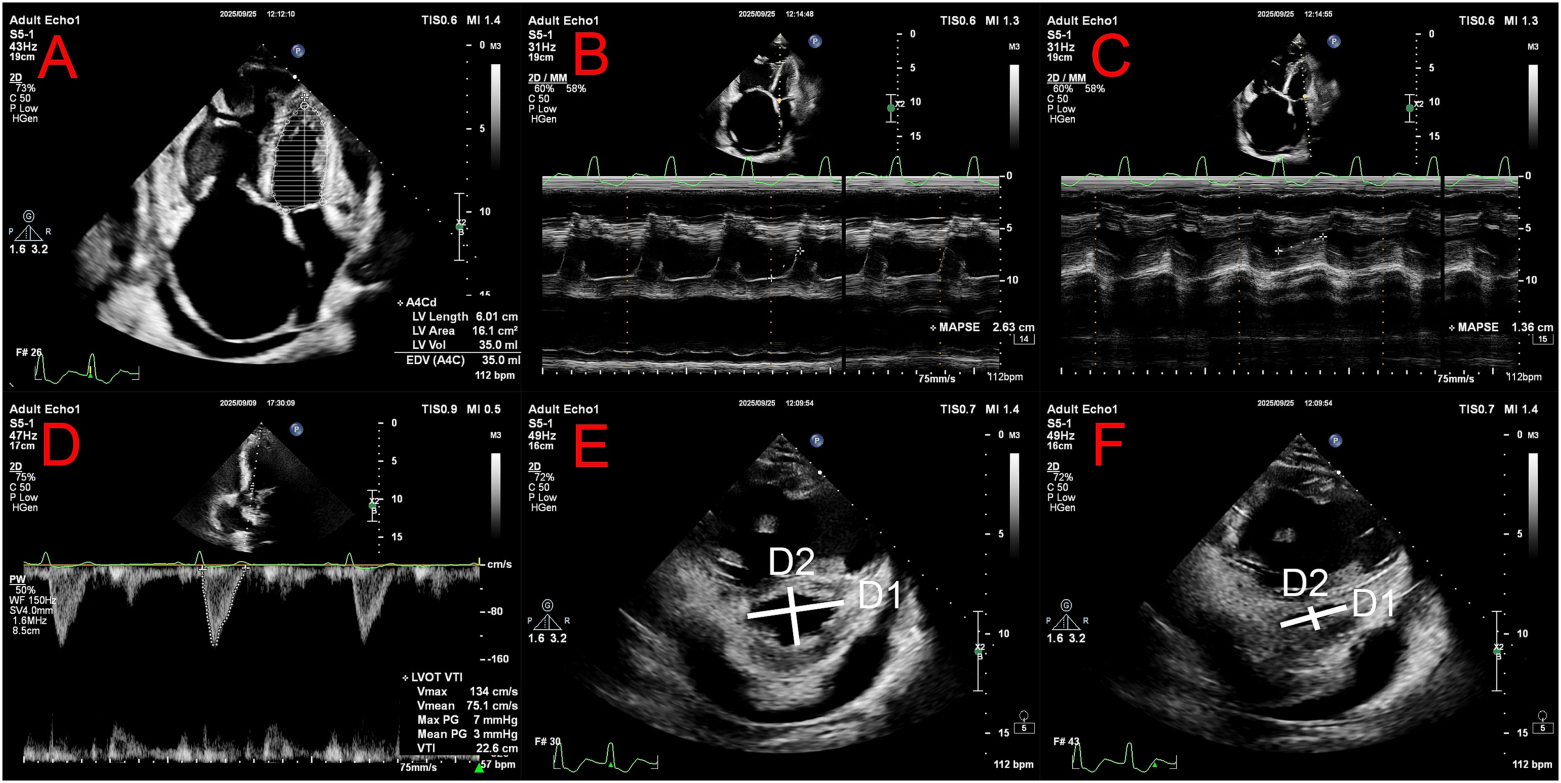
Assessment of left ventricular in pulmonary hypertension. **(A)** Left ventricular end-diastolic volume measurement in the apical four chamber view. **(B)** Septal MAPSE measurement in the apical four chamber view. **(C)** Lateral MAPSE measurement in the apical four chamber view. **(D)** The velocity time integral of the left ventricular outflow tract measured by pulsed-wave Doppler. Eccentricity index, the ratio of the diameters (from compact myocardium) parallel (D1) and perpendicular (D2) to the ventricular septum, as measured at end diastole **(E)** and end systole **(F)**.

The expansion of the right ventricle in patients with PH is intrinsically linked to the compression and under-filling of the left ventricle, resulting in increased ventricular stiffness and impaired diastolic function (13). Typically, this LV under-filling is associated with invasive hemodynamic measurements, a leftward shift of the interventricular septum, and prolonged right ventricular systole, all of which may detrimentally affect clinical outcomes in PH (12, 31). Left ventricular volume parameters serve as valuable indicators that provide direct information about the LV filling status. A decrease in left ventricular end-diastolic volume (LVEDV) to ≤40 mL/m^2^ indicates a deficit in LV filling, correlating with diminished survival rates in patients with PH and serving as an independent predictor of mortality (Figure 3) (32). In a relevant meta-analysis, it was demonstrated that for every 1 mL/m^2^ reduction in the left ventricular end-diastolic volume index, the risk of mortality increases by 1.8%, without a corresponding rise in clinical deterioration (33). Recent studies have revealed that LVEDV is associated with the RV-pulmonary artery (PA) coupling, quantified via the end-systolic elastance to arterial elastance ratio (Ees/Ea) derived from pressure-volume loops. The observation of reduced LVEDV in instances of severe RV-PA uncoupling (Ees/Ea < 0.8) suggests that such uncoupling may impair left heart function due to compromised LV preload (34). Nevertheless, the additional utility of LVEDV in refining risk stratification for patients with PH remains to be established, necessitating further investigation.

There is also growing interest in the assessment of LV pressure among patients with PH, with ongoing efforts to utilize echocardiography and Doppler techniques to predict increases in left ventricular end-diastolic pressure (LVEDP) in individuals with PH (35). As a widely recognized surrogate for LV filling pressure, the E/e′ ratio measured by tissue Doppler imaging (TDI) has been shown to reliably predict the pulmonary capillary wedge pressure (PCWP) in patients with IPAH and can reflect improvements in LV filling following long-term targeted therapy (36). From a phenotype-specific perspective, parameters reflecting LV filling pressure, including LVEDP and E/e′, are particularly valuable for differentiating PH subtypes. Elevated values are more consistent with post-capillary PH due to left heart disease, whereas in pre-capillary PH these parameters are typically normal or reduced, reflecting decreased LV preload rather than intrinsic diastolic dysfunction. In cases of chronic thromboembolic pulmonary hypertension (CTEPH), the average pulmonary artery wedge pressure (mPAWP) and LVEDP are employed to evaluate left ventricular filling pressure (LVFP). Notably, although an increase in LVFP adversely influences the long-term prognosis of patients with CTEPH, this is likely attributed to intrinsic left heart conditions (37). Cardiac magnetic resonance (CMR) imaging of pulmonary vein area (PVA) and flow may elucidate the physiological aspects of LV filling in pulmonary arterial hypertension (PAH) by distinguishing between elevated LVFP and true LV under-filling. Patients with PAH frequently exhibit diminished S wave velocity in the pulmonary veins, reduced left ventricular volume, and attenuated function. Therefore, it can be inferred that left ventricular dysfunction in PAH predominantly results from inadequate filling rather than intrinsic myocardial disorders (38). This distinction underscores the importance of interpreting LV abnormalities within specific PH phenotypes, as similar functional changes may arise from fundamentally different mechanisms. However, the application of pulmonary venous flow patterns in PH lacks sufficient broad validation. In this context, it should serve as an adjunctive reference for LV filling status rather than a definitive diagnostic indicator. Conversely, the left ventricular intraventricular pressure gradient (LV-IVPG) is emerging as a sensitive parameter for detecting subtle mechanical changes in the LV, especially since left ventricular ejection fraction (EF) tends to remain stable in patients with pre-capillary PH (PPH). This stability may indicate early alterations in diastolic function that influence LV performance (39). However, the clinical relevance of such parameters may vary across PH subtypes and should be interpreted in the context of the underlying hemodynamic phenotype.

Beyond intrinsic LV parameters, the size and function of the left atrium (LA) are increasingly recognized as sensitive barometers of LV diastolic dysfunction and elevated filling pressures. In addition to echocardiographic assessment, recent studies utilizing automated 3D volumetry based on CT pulmonary angiography have demonstrated that LA volume is superior to traditional linear indices in identifying left heart disease-associated PH (40). This highlights the potential role of CT as a complementary modality in the assessment of left heart involvement in PH. Furthermore, LA strain assessed by CMR can be utilized to detect early left heart involvement in PH; specifically, impaired LA reservoir function is considered a highly sensitive indicator, potentially outperforming certain traditional LV functional parameters (41). Importantly, left atrial structural and functional changes are particularly informative for identifying post-capillary PH, where chronically elevated filling pressures directly drive atrial remodeling.

Moreover, the profound consequence of adverse ventricular interaction—LV underfilling—can be quantitatively assessed through a novel marker derived from transthoracic echocardiography: the LV volume-to-mass (V/M) ratio. This index reflects the mismatch between chamber volume and myocardial mass, emerging as a valuable prognostic indicator in PAH that demonstrates incremental value when incorporated into traditional combined risk models (42). This integration offers enhanced prognostic capabilities and may serve as a crucial tool in optimizing the comprehensive care of patients with PAH. In contrast, in pre-capillary PH, LV underfilling primarily reflects reduced preload secondary to RV dysfunction, further emphasizing the importance of phenotype-specific interpretation.

Overall, the assessment of LV diastolic function in PH extends beyond the identification of abnormal filling patterns. Its primary clinical value lies in differentiating pre-capillary from post-capillary PH. In pre-capillary PH, these parameters predominantly reflect ventricular interdependence and reduced LV preload, whereas in PH-LHD they indicate intrinsic LV dysfunction and elevated filling pressures. Therefore, integrating these indices within a phenotype-specific framework is essential for accurate diagnosis and clinical decision-making.

## 5 Assessment of left ventricular systolic function

As previously noted, severe PH often leads to dilation of the right ventricle and right atrium, abnormal motion of the interventricular septum, compression of the left ventricular cavity, and inadequate LV filling. These abnormalities directly lead to a reduction in stroke volume (SV), cardiac output (CO), and left ventricular ejection time (LVET) (43, 44). The left ventricular stroke volume index (LVSVI) is valuable for predicting major adverse cardiovascular events (MACE) in patients with PH and provides prognostic information related to PH (45, 46). Thus, it is essential to measure CO individually. This can be systematically assessed in patients with PAH using transthoracic echocardiography, alongside measurements of the left ventricular outflow tract (LVOT) diameter and the velocity-time integral (VTI) recorded via pulsed-wave Doppler. The velocity-time integral of the LVOT is a practical alternative for predicting poor long-term survival in patients with PH. It also serves to evaluate the severity of PAH and predict long-term prognosis (Figure 3) (47). As the stages of PAH progress, there is a notable decrease in both left ventricular diameter and wall thickness, although these changes typically manifest only in the later stages of the disease (26).

The left ventricular ejection fraction (LVEF) may remain normal in patients with PH; thus, it does not fully reflect changes in LV systolic function. Over the past two decades, myocardial strain has emerged as a more sensitive parameter for assessing LV systolic function than LVEF, as LV systolic strain may decrease even when LVEF remains stable. At present, LV strain is increasingly acknowledged as a complementary and alternative measure to LVEF in the evaluation of PH (48, 49). Analysis of both global and regional strain in the LV of patients with PH has revealed relationships with RV systolic function and hemodynamics (50). This not only provides evidence of interacting ventricular functions during the systolic phase of PH but also facilitates early detection of subclinical LV dysfunction through quantitative assessment (51). Recent studies have highlighted the prognostic value of global longitudinal strain (GLS) in various subtypes of PH. For instance, in patients with severe PAH and normal LVEF, LVGLS shows an independent association with mortality (52). Measurements of LVGLS using cardiac magnetic resonance feature tracking (CMR-FT) have proven to be reliable indicators of mortality prediction in patients with precapillary PH (53). Furthermore, analysis of LVGLS has indicated an increased overall mortality in patients with systemic sclerosis-related PH (SSc-PH) (54). However, the significance of LV strain in risk stratification for PH may not have been fully appreciated, and future studies should further explore the potential of LV strain imaging in accurately identifying subclinical LV dysfunction and assessing risk in patients with PH. This could provide a more comprehensive understanding of LV performance (see Table 1).

**Table 1:** Parameters reflecting left ventricular function in pulmonary hypertension.

Parameter	Modality	Relevant condition	Reflects	Significance	Ref.
LVEDV	Echo/CMR/CT	IPAH	LV preload and filling status	Reduced survival rate, an independent predictor of mortality	(32)
		PH		RV-PA uncoupling may affect LV function by impairing LV preload	(34)
LV V/M ratio	Echo	PAH	LV preload and filling status	Reflects ventricular underfilling and maladaptive remodeling; provides incremental prognostic value in combined risk models.	(42)
E/e′	Echo	IPAH	LV filling pressure	Reliably predicts elevated PCWP and reflects filling improvement post-therapy	(36)
LVEDP	Invasive hemodynamics	PH	LV filling pressure		(35)
LVFP	Invasive/echo	CTEPH	LV Filling Pressure	Reduced survival rate	(37)
LA volume	Echo/CMR/CT	PH-LHD	LV filling pressure	Identifying post-capillary hemodynamic	(40)
LA strain	Echo/CMR	PH-LHD	LV filling pressure	Detecting early left heart involvement	(41)
Pulmonary vein area and flow	Echo/CMR	PAH	LV preload and filling status	Possible LV dysfunction due to inadequate filling	(38)
LV-IVPG	CMR	pPH	Impaired diastolic function	Impaired LV diastolic function	(39)
LVSVI	Echo/CMR	PH	LV systolic function	Independent predictor of MACE/prognosis	(45)
		PAH			(46)
TVI-LVOT	Echo	PAH	LV systolic function	Noninvasive PAH severity assessment and outcome prediction	(47)
LVGLS	Echo/CMR	sPAH	LV systolic function	Independently associated with mortality in severe PAH	(52)
		pPH		Reliable predictor of mortality in pre-capillary PH	(53)
		SSc-PH		Useful for LV dysfunction detection and risk assessment in SSc-PH	(54)
LV torsion rate	CMR	Pediatric PAH	Interventricular dependence and systolic performance	Reduced LV torsion rate strongly associates with diminished RV-Ees	(14, 57)
IVS shape	Echo/CMR	PH	Interventricular dependence and mechanical compression	IVS shape changes correlate with ventricular dysfunction in PH	(61)
EI	Echo/CMR	Pediatric PH	Interventricular dependence and mechanical compression	EI accurately identifies clinically significant PH	(62)
SSI	CMR	PH	Interventricular dependence and mechanical compression	Reduced SSI reliably detects PH	(63)
IVS angle	CMR	Cpc-PH	Interventricular dependence and mechanical compression	Elevated systolic IVS angle predicts mortality-risk PH from left heart disease	(65)
Septal strain	Echo/CMR	PH	Interventricular dependence and regional systolic performance	Ventricular interdependence contributes to LV dysfunction in PH	(67)
RV/LV end-diastolic diameter ratio	Echo/CMR/CT	IPAH	Global interventricular dependence	RV/LV ratio reflects IPAH severity and independently predicts prognosis	(70)
RV/LV end-systolic ratio	Echo/CMR/CT	Pediatric PAH	Global interventricular dependence	End-systolic RV/LV ratios outperform TAPSE for assessing global RV systolic dysfunction	(73)
RV/LV diameter ratio	Echo/CMR/CT	ILD-PH	Global interventricular dependence	Elevated CT-derived RV/LV ratio noninvasively stratifies risk in ILD-PH	(74)
RV/LV dimension ratio	Echo/CMR/CT	Pediatric PH	Global interventricular dependence	RV/LV ratios correlate with functional class, hemodynamics, and NT-proBNP	(75)
LVEDP/RVEDP	Invasive hemodynamics	PH	Global interventricular dependence	LVEDP/RVEDP ratio predicts treatment response in PH, independent of standard severity markers	(76)
SSF, DRF	Echo/CMR	Pediatric PAH	Ventricular interdependence	Greater dyssynchrony in PAH predicts worse outcomes and precedes LV dysfunction	(78)
LV intra-ventricular dyssynchrony	Echo/CMR	IPAH	Ventricular electromechanical synchrony	Intraventricular synchrony correlates with disease severity markers	(80)

Left ventricular torsion plays a crucial role in cardiac systolic and diastolic functions. During systole, torsional movements contribute to ventricular ejection; during diastole, the untwisting motion of the ventricle releases the elastic potential energy stored during twisting, thereby facilitating ventricular filling. This can be evaluated through echocardiography and cardiac magnetic resonance imaging (55–57). Indeed, left ventricular torsion indirectly reflects the geometric changes in the right and left ventricles associated with PH. During PH or right ventricular failure, left ventricular torsion decreases and is delayed, along with the flattening of the interventricular septum and increased left ventricular eccentricity (55, 56). Furthermore, the relationship between left ventricular torsion rate and right ventricular systolic function has been validated in animal models and pediatric patients with PH. Quantitative analysis of the reduction in left ventricular torsion rate has demonstrated a close correlation with invasive measurements of end-systolic elastance (14, 57). This further emphasizes the critical role of inter-ventricular mechanical dependence and its correlation with the decline in RV function in PH.

In contrast, the mitral annular plane systolic excursion (MAPSE), akin to the tricuspid annulus plane systolic excursion (TAPSE), is a widely utilized, simple M-mode echocardiographic parameter commonly employed to assess the longitudinal systolic function of the left ventricle. A meta-analysis has indicated that MAPSE offers more reliable, robust, and user-friendly clinical value in diagnosing, predicting, and treating heart failure compared to EF or GLS, allowing for rapid assessment of left ventricular systolic function (Figure 3) (58). However, the potential value of MAPSE in assessing LV function in PH has yet to be fully realized, and further research is needed to validate this, aiding in the identification of additional simple and reliable indicators for evaluating LV function in PH.

## 6 Assessment of the interventricular septum

In PH, the right heart impacts left ventricular (LV) function through the interventricular septum (IVS), which protrudes into the LV throughout the cardiac cycle, exhibiting abnormal septal motion. Recently, the septum has emerged as the most critical mediator of RV influence on LV function in PH, illustrating the intricate interaction between the right and left ventricles in patients with PH. The degree of distortion in septal shape can be quantified and is closely associated with pulmonary artery systolic pressure (PAP) (59). These characteristics convey an important message: the assessment of left ventricular function must be understood in the context of analyzing the shape and dynamic changes of the interventricular septum and its impact on ventricular function, particularly in patients with PH, as a means of evaluating disease progression or regression (60). When analyzing septal shape in PH patients via CMR, differences in ventricular function were observed among PH patients with varying IVS shapes; greater degrees of IVS deformation correlate with diminished left heart function (61). This IVS deformation can be quantified using the eccentricity index (EI), allowing for the accurate identification of clinically significant PH patients (Figure 3) (62). This is particularly noteworthy, as this parameter has demonstrated considerable advantages in assessing PH at baseline and during subsequent follow-up. Crucially, the clinical interpretation of the EI and septal flattening must remain phenotype-specific. In pre-capillary PH, these geometric alterations reliably capture the extent of RV pressure overload and mechanical LV compression. However, their diagnostic specificity diminishes in post-capillary PH. In left heart disease (Group 2), intrinsic LV remodeling—such as hypertrophy or volume expansion—often counteracts the right-to-left septal shift. Consequently, classic D-shaped geometric alterations may be absent in these patients despite significantly elevated pulmonary pressures, dictating that geometric indices must always be evaluated alongside comprehensive hemodynamic data.

Additionally, the swing of the interventricular septum has recently garnered attention and is frequently observed in CMR cine sequences of patients with PH. The septum swing index (SSI) is an emerging, simple, and reliable parameter that combines left and right heart interactions, demonstrating good sensitivity and specificity in detecting PH. In patients with PH, a lower SSI serves as an independent predictor of the disease, which can be used in conjunction with PH risk stratification for prognostic assessment (63). However, future large-scale prospective studies are needed to further validate this and to identify the optimal threshold for differentiation. Furthermore, previous research has highlighted the potential significance of the interventricular septal angle. The interventricular septal angle is measured as the angle between the intersection points of the septal insertion points in CMR and the midpoint of the interventricular septum, which typically exhibits an increased angle in patients with PH, serving as an indicator of left ventricular septal deviation due to right ventricular hypertension (64). It has been shown to correlate with the severity of PH and can be used for non-invasively identifying cohorts of suspected PH patients and those with elevated pulmonary artery wedge pressure (PAWP), who present with both precapillary and postcapillary PH, as well as for predicting all-cause mortality in patients with PH secondary to left heart diseases (65). In summary, this emerging parameter is particularly important for identifying patients and facilitating timely interventions; however, its clinical application requires further validation.

Indeed, when measuring regional myocardial strain of the left ventricle in PH, the effect of RV pressure overload on the IVS is greater than on the LV free wall (50, 66). Therefore, it is essential to focus on the myocardial strain of the interventricular septum, as this not only indicates left heart failure in patients with PH occurring significantly earlier than reductions in LVEF (67) but also reflects right heart dysfunction in patients with PH (68). Future research should focus on utilizing CMR and strain imaging techniques to thoroughly investigate the morphological and functional changes of the interventricular septum in PH. Further validation of the septal swing index and septal angle as indicators for the diagnosis and prognosis of PH is warranted.

## 7 Comprehensive assessment of left–right ventricular function

Previous studies have primarily evaluated left or right ventricular function in isolation. In recent years, awareness has grown that certain indicators incorporating information from both left and right ventricles play an increasingly important role (69). The ratio of RV to LV diameters (RV/LV) can be easily obtained non-invasively and reflects the interaction between the ventricles under right ventricular pressure load, including leftward interventricular septum displacement and RV dilation. This ratio encompasses RV failure, remodeling, adverse hemodynamics, and resultant LV compression (70). In pediatric PH, the RV/LV ratio is frequently associated with poorer invasive hemodynamics, higher NT-proBNP levels, and various adverse clinical events (71). A ratio greater than 1 indicates an increased risk of mortality, prompting closer monitoring and management of pediatric patients with PH (72). Comprehensive assessments indicate that the RV/LV ratio is superior to TAPSE for evaluating overall right ventricular systolic dysfunction. The RV/LV end-systolic ratio tends to normalize in children with severe pulmonary arterial hypertension after lung transplantation (LuTx), making it valuable for evaluating cardiac function post-LuTx for severe PAH (73). However, despite initially promising results, the role of the RV/LV ratio in adult PH remains uncertain. Substantial evidence indicates that an elevated RV to LV diameter ratio measured by CT pulmonary angiography (CTPA) carries significant prognostic information. This ratio acts as an independent predictor for patients with PH who have interstitial lung disease (ILD), providing a simple, non-invasive method for risk stratification in suspected ILD-PH patients (74). Furthermore, the volumetric RV/LV ratio offers substantial value independent of existing markers of PH severity when compared to diameter measurements. In terms of diagnostics, combining the RV/LV volumetric ratio with indexed RV end-diastolic volume (RVEDV) improves detection rates of RV enlargement in patients with PAH (75). This finding may provide a more comprehensive assessment of RV size. When evaluating the response to pharmacological therapy, the ratio of left ventricular end-diastolic pressure to right ventricular end-diastolic pressure (LVEDP/RVEDP) reflects complex interactions between ventricular remodeling and maladaptation occurring in chronic PH, emerging as a new marker for the responsiveness of PH patients to pulmonary vasodilatory therapy (76). Crucially, while biventricular metrics like the RV/LV ratio offer a holistic view of cardiac adaptation, their clinical interpretation must remain strictly phenotype-driven. A reduced LV size in pre-capillary PH is a direct consequence of mechanical underfilling. In stark contrast, in PH-LHD, such dimensional changes often coexist with—or are masked by—intrinsic pathological remodeling. Therefore, combined ventricular indices should not be interpreted in isolation; rather, they serve as powerful complementary tools within a broader phenotypic assessment.

Despite promising preliminary evidence, large-scale prospective validation is necessary before routine clinical application. Further research on indicators such as the RV/LV ratio and LVEDP/RVEDP is warranted in the context of PAH risk stratification and treatment response assessment to address the limitations of current studies.

## 8 Assessment of ventricular dyssynchrony

Due to the unique pathophysiology of PH, elevated RV pressure load in advanced stages of PH leads to RV dysfunction, as evidenced by increased isovolumetric contraction, prolonged contraction duration, and increased isovolumetric relaxation (77). Ultimately, this results in prolonged RV contraction periods and shortened RV relaxation periods, significantly diminishing the contractile efficiency of the RV (prolonged contraction yet reduced ejection volume) (7). This continuous response, characterized by the disparate timing of RV and LV events, is a principal determinant of interventricular dyssynchrony and leftward deviation of the interventricular septum, leading to insufficient LV filling and a subsequent reduction in stroke volume (24).

Indeed, apart from the detrimental effects of ventricular-ventricular interaction that leads to interventricular dyssynchrony, RV-induced LV discoordination may play a critical role in the causal pathway from PH to clinically significant LV dysfunction. From a mechanistic perspective, this discoordination is driven by a triad of factors: (1) Electrical dyssynchrony (E-dys): manifesting as widened QRS complexes; (2) Regional myocardial strain heterogeneity and (3) Prolonged RV mechanical contraction (78, 79). From a clinical standpoint, ventricular dyssynchrony has the potential to act as an indicator of disease severity. Indicators of diastolic dyssynchrony, obtained noninvasively from CMR imaging, demonstrate increased electrical and mechanical desynchronization of the LV under conditions of PH/RV failure, which may precede overt LV functional impairment (78). This suggests an important implication: enhancing the analysis of ventricular synchronization could yield clinical benefits. Jayasekera and his colleagues further analyzed within-ventricle dyssynchrony of the LV, finding it to correlate with disease severity in patients with IPAH, potentially offering additional prognostic value (80).

The recognition of this mechanistic framework also carries significant therapeutic implications. Although Cardiac Resynchronization Therapy (CRT) has been established as an effective treatment in left heart failure, its role in PH remains to be fully elucidated (81). Theoretically, improving interventricular timing or enhancing ventricular coordination could help alleviate adverse ventricular interactions and improve overall cardiac function. Furthermore, ventricular dyssynchrony may not only serve as a prognostic indicator but also has the potential to act as a reversible therapy-response marker.

Crucially, the clinical interpretation of ventricular dyssynchrony must remain strictly phenotype-dependent. In pre-capillary PH, LV discoordination is predominantly a mechanical consequence of RV pressure overload and adverse interventricular interaction. Conversely, in PH due to left heart disease, dyssynchrony typically stems from intrinsic myocardial pathology or primary LV conduction defects. Recognizing these divergent mechanistic origins is essential, as they will fundamentally dictate future targeted therapeutic strategies.

## 9 Future and perspectives

Although the clinical relevance of left ventricular (LV) assessment in pulmonary hypertension (PH) is increasingly recognized, several important gaps remain.

First, there is a clear need for phenotype-specific imaging biomarkers. Most currently used parameters lack sufficient specificity to distinguish between different PH subtypes. Emerging indices—such as the septal swing index (SSI), interventricular septal angle, LV intraventricular pressure gradient (LV-IVPG), and the LV volume-to-mass (V/M) ratio—are promising, but require further validation. In particular, defining clinically meaningful thresholds for differentiating pre-capillary from post-capillary PH, especially at early stages, remains a key priority.

Second, standardization of LV assessment is urgently needed. Substantial heterogeneity persists in measurement techniques, cut-off values, and reporting strategies across studies, which limits clinical applicability. Future efforts should focus on establishing unified protocols, including standardized acquisition methods, reference ranges, and phenotype-oriented interpretation frameworks.

Third, the integration of multimodality imaging represents a major opportunity. In clinical practice, a complementary approach is likely to be most effective, with echocardiography serving as the first-line modality, cardiac magnetic resonance providing detailed structural and functional characterization, and computed tomography offering accurate volumetric assessment. Looking ahead, the incorporation of artificial intelligence may enable the integration of these data into composite indices—such as a “ventricular interdependence score”—to improve diagnostic precision and risk stratification.

Fourth, large-scale prospective studies are needed to strengthen the evidence base. Much of the current knowledge is derived from small, single-center or cross-sectional studies. Well-designed multicenter cohorts with long-term follow-up, including diverse etiologies such as interstitial lung disease-associated PH and systemic sclerosis-associated PH, will be essential to validate the independent prognostic value of LV-related parameters.

Finally, the role of LV assessment in guiding therapy remains insufficiently explored. Future work should clarify whether ventricular dyssynchrony can serve as a therapeutic target—for example, in the context of cardiac resynchronization strategies—and whether LV remodeling, particularly atrophic changes, is reversible with targeted PH therapies.

## 10 Conclusion

The impact of pulmonary hypertension on the left ventricle extends well beyond passive mechanical compression. Through adverse ventricular interdependence, right ventricular (RV) pressure overload initiates a cascade of LV alterations, including reduced preload, septal deformation, and mechanical dyssynchrony. In advanced stages, these changes may progress to intrinsic atrophic remodeling of the LV myocardium.

Several key conclusions emerge from this review.

First, the principal clinical value of LV assessment lies in phenotype differentiation. In pre-capillary PH, LV abnormalities primarily reflect reduced filling and mechanical constraint secondary to RV overload. In contrast, in PH due to left heart disease, these findings indicate intrinsic myocardial dysfunction. A phenotype-oriented interpretation is therefore essential for accurate diagnosis.

Second, multiple LV-related parameters demonstrate independent prognostic significance. These include reduced LV end-diastolic volume, impaired global longitudinal strain, altered LV geometry, and emerging indices such as the LV V/M ratio and septal-derived markers. Integrating these parameters into multiparametric models may enhance risk stratification.

Third, the left atrium provides important complementary information. Changes in left atrial size and function reflect chronic alterations in LV filling pressures and may be particularly useful in identifying post-capillary PH at an early stage.

Finally, ventricular dyssynchrony represents an underrecognized but potentially modifiable feature. It may precede overt systolic dysfunction and could serve as both a marker of disease severity and a potential indicator of treatment response.

Taken together, LV assessment in PH is evolving from a descriptive adjunct to a central component of clinical evaluation. A framework that integrates phenotype-specific interpretation with multimodal imaging will be essential to fully realize its diagnostic and prognostic potential.

## Glossary

LVEDV: left ventricular end-diastolic volume
LVEDP: left ventricular end-diastolic pressure
LVFP: left ventricular filling pressures
LV-IVPG: LV intraventricular pressure gradients
LVSVI: indexed right ventricular end-systolic volume
TVI-LVOT: time-velocity integral of the left ventricular outflow tract
LVGLS: LV global longitudinal strain
IVS shape: shape of Interventricular Septum
EI: eccentricity index
SSI: septum swing index
IVS angle: interventricular septal angle
RV/LV dimension ratio: right ventricular /left ventricular dimension ratio
LVEDP/RVEDP: the ratio of left ventricular end-diastolic pressure to right ventricular end-diastolic pressure
SSF、DRF: systolic stretch fraction/diastolic relaxation fraction;
IPAH: idiopathic pulmonary arterial hypertension
CTEPH: chronic thromboembolic pulmonary hypertension
PAH: pulmonary arterial hypertension
pPH: precapillary pulmonary hypertension
sPAH: severe pulmonary arterial hypertension
SSc-PH: systemic sclerosis-associated pulmonary hypertension
Cpc-PH: combined pre- and post-capillary pulmonary hypertension
ILD-PH: interstitial lung disease-associated pulmonary hypertension
RV-PA uncoupling: right ventricular-pulmonary arterial uncoupling
MACE: major adverse cardiovascular events
Ees: end-systolic elastance.
